# Demographic relevancy of increased use of assisted reproduction in European countries

**DOI:** 10.1186/1742-4755-11-37

**Published:** 2014-05-26

**Authors:** Jirina Kocourkova, Boris Burcin, Tomas Kucera

**Affiliations:** 1 Department of Demography and Geodemography, Faculty of Science, Charles University in Prague, Albertov 6, Prague, Czech Republic

**Keywords:** Fertility, Childbearing postponement, Assisted reproduction, European countries

## Abstract

**Background:**

Delayed childbearing in European countries has resulted in an increase in the number of women having children later in life. Thus more women face the problem of age-related infertility and cannot achieve their desired number of children. Childbearing postponement is one of the main reasons for the increasing use of assisted reproductive technology (ART) and conversely, the latter may be one of the factors contributing to the rise in female childbearing age. The research goal of our article is to evaluate the demographic importance of ART increased use and to examine its impact on both the fertility rate and birth timing.

**Methods:**

Comparative analysis based on demographic and ART data collected by the European IVF-monitoring (EIM) Consortium for the European Society of Human Reproduction and Embryology (ESHRE).

**Results:**

Most countries with a higher total fertility rate (TFR) also registered a higher number of treatment cycles per 1 million women of reproductive age. Despite the positive relationship between the postponement rate and the demand for ART among women aged 35 and older, the highest share of children born after ART was not found in countries characterized by a “delayed” fertility schedule. Instead, the highest proportion of ART births was found in countries with fertility schedules concentrated on women aged between 25 and 34. Accordingly, the effective use of ART can be expected in populations with a less advanced postponement rate.

**Conclusions:**

ART can have a demographic relevancy when women take advantage of it earlier rather than later in life. Furthermore it is suggested that the use of ART at a younger age increases women’s chance of achieving their reproductive goals and reduces the risk of age-related infertility and failed ART. Based on a demographic approach, reproductive health policy may become an integral part of policies supporting early childbearing: it may keep women from delaying too long having children and increase the chance of diagnosing potential reproductive health problems requiring a timely ART application.

## Background

The reproductive behaviour of most Europeans has shifted from an early to a late or very late childbearing pattern [1-4]: at present women start to build a family into their 30s or even later. In 2010 the mean age of mothers at first childbirth was between 28 and 30 years in EU member states [5]. On one hand, delayed childbearing has numerous advantages as people may be more mature and considerate when they start a family [4]. On the other hand, there are several severe drawbacks that need to be addressed and studied: the longer the wait, the higher the risk of negative health outcomes (for mother and child), the higher the risk of having to rely on assisted reproductive technology (ART) - mainly in vitro fertilisation (IVF) - and the higher the risk of not achieving optimal family size.

Starting childbearing later means having less time to achieve reproductive goals since biological limits of childbearing have not shifted to later ages. In addition, an increasing part of reproductive plans is implemented at the age of female fecundity decrease which may be reflected in a perceived delay, a difficulty of conceiving or carrying a baby to term [6]. Thus childbearing postponement has become an issue related to female reproductive health and is considered to be the main factor of ART increasing use in countries in question [7]. At the same time age-related subfertility is often considered to be a problem easily solved by the application of ART though it may not make up for all lost births due to the natural decline in fertility after the age of 35 [8].

Childbearing postponement is increasingly relevant to demographic trends. Although most European countries have registered a similar decrease in fertility rates of young women, they significantly differ by the intensity of fertility recuperation, i.e. compensatory increase in fertility rates of women at a higher age above 30 [9]. As a result, a large cross-country variation in current fertility rates exists [5]. Accordingly, there is a tendency to reconsider public policies related to fertility and assisted reproductive technology (ART) support at national levels as well as within the EU: ART policies have definitely become much discussed topics [10]. Moreover ART treatments have generated important policy questions regarding their cost-effectiveness and safety [11].

ART use widely varies among European countries: despite the fact that some European countries do not provide complete statistics on ART, in 2009 the average number of treatment cycles per 1 million inhabitants ranged from 166 in Moldova to 2726 in Denmark [12]. Since it is rather improbable that countries would significantly differ regarding the share of infertile couples within their populations, the wide range in ART use may result from an unequal access to it [13,14]. It was estimated that while circa 3000 couples per 1 million inhabitants may be eligible for ART, only half of them do request it [15,16]. Provided that each couple needs on average more than one treatment cycle, the real need would exceed 2500 cycles. In 2009, Denmark, Iceland, and Belgium ranked above this estimated number while other European countries ranked well below it. Scandinavian countries met levels of utilization that approximated demand [13] while most European countries have probably not met the rising need for ART yet. However financial, medical, psychosocial, moral or ethical grounds may distinctly affect the use of infertility treatments in compared countries [17].

The core of our paper is not a mere cross-country comparison of ART utilization but does focus on searching for a relationship between the increased use of ART and fertility trends. While the impact of childbearing postponement on the fertility rate and ART demand has been analysed and discussed [18,10-21], the impact of ART increasing use on both the fertility rate and birth timing has not been studied comprehensively. Our research goal was to evaluate the demographic potential of ART increasing use in relation to childbearing postponement. Consequently ART higher use is expected in countries with a higher fertility rate and a delayed fertility pattern. Nevertheless findings are interpreted with caution as both the fertility rate and birth timing are influenced by additional individual and social factors that may play a more significant role.

## Methods

The cross-national comparison of available data on ART use in Europe has been collected by the European IVF-monitoring (EIM) Consortium for the European Society of Human Reproduction and Embryology (ESHRE) since 1997 and published in Human Reproduction [22-26,12]. Although data on ART have been collected by national registers, the method of reporting varied among countries and some did not provide complete information. It is particularly pertinent as to live births following the use of ART due to difficulties when gathering pregnancy outcomes: only countries with a 100% coverage - i.e. including all clinics comprehensive participation - were included in the analysis and are presented in Figures, except Table 1. In Table 1, countries with a low rate of incompleteness were taken into account as well. Table 1 provides information on structure of women treated with IVF/ICSI by age groups while Figures present the level of ART use where complete data are essential for full comparability. ART includes all forms of treatments and techniques related to the in vitro handling of both human oocytes and sperm, or embryos, for the purpose of creating a pregnancy [27]. ART does not include assisted insemination. Available tables covered data on IVF (in vitro fertilization), ICSI (intracytoplasmic sperm injection), FER (frozen embryo replacement), ED (oocyte donation), IVM (in vitro maturation), PGD (preimplantation genetic diagnosis), and FOR (frozen oocyte replacement).

**Table 1:** Age structure of women treated with IVF/ICSI in selected European countries, 1997 and 2009

**Country**	**1997**			**Country**	**2009**		
	**IVF + ICSI (%)**				**IVF + ICSI (%)**		
	**≤34**	**35-39**	**≥40**		**≤34**	**35-39**	**≥40**
Czech Republic	63.5	27.0	9.4	Czech Republic^b^	67.2	25.4	7.4
Denmark	NA	NA	NA	Denmark	50.6	31.0	18.4
Finland	57.9	27.9	14.1	Finland	55.0	31.6	13.5
France	58.3	29.7	12.0	France	NA	NA	NA
Germany	55.7	29.9	14.5	Germany^c^	45.5	41.2	13.3
Hungary^a^	66.7	22.5	11.0	Hungary	56.3	30.1	13.6
Iceland	40.8	29.6	14.7	Iceland	NA	NA	NA
Ireland	NA	NA	NA	Ireland^a^	30.8	47.2	22.0
Italy	54.3	33.2	12.5	Italy	31.3	40.5	28.2
Portugal	63.3	30.4	6.3	Portugal	49.1	39.0	12.0
Slovenia	NA	NA	NA	Slovenia	51.9	32.8	15.4
Spain	50.3	38.6	11.2	Spain^a^	40.9	45.9	13.2
Sweden	56.5	34.0	9.5	Sweden	49.0	39.0	11.9
Switzerland	49.1	36.1	14.8	Switzerland^a^	36.6	42.8	20.5
United Kingdom	54.4	32.9	12.7	United Kingdom	41.9	40.9	17.2

^a^Countries with partial data coverage – Reporting IVF clinics in the country/Total IVF clinics in the country. Hungary – 6/7, Ireland – 6/7, Spain – 109/166, Switzerland – 25/26.

^b^2006.

^c^2007.

Differences in ART use among countries were analysed in relation to data on fertility [28]. As data on ART births according to female age were not available, we could merely use general indicators such as proportion of ART births, number of treatment cycles per 1 million women of reproductive age, and proportion of women treated by IFV/ICSI according to age groups. In order to analyse the relationship between increased ART use and fertility trends, total fertility rate (TFR) and fertility postponement index (FPI) counted from all births in a given country, were taken into account. However we were aware of shortcomings of such an approach as both the fertility rate and the postponement rate are influenced by individual factors as well as external factors such as cultural values, economic factors or family policies [10]. Although merely used for a descriptive purpose, some common tendencies regarding the link between ART use and fertility trends could be identified across countries. TFR is defined as the average number of live births per woman during her lifetime, as if she were to pass through all her childbearing years conforming to age-specific fertility rates (ASFR) of a given year [29]. ASFR were used to create fertility age schedules for selected countries enabling us to identify countries with a “delayed” fertility schedule: the greatest concentration of births occurring in early 30s and a considerable amount of births occurring in late 30s. Lesthaeghe & Niedert´s fertility postponement index (FPI) is the ratio of the sum of ASFR above age 29 to the sum of these rates between ages 20 and 29 [30,31]. FPI was constructed to measure the postponement rate in a simplified way. FPI of about 1.3 and more, identifies countries with an advanced postponement rate, i.e. “delayed fertility pattern” while FPI at 0.8 and lower, identifies countries with a low postponement rate, i.e. “early” fertility pattern.

## Results

### Trends in ART use

**Figure 1:**
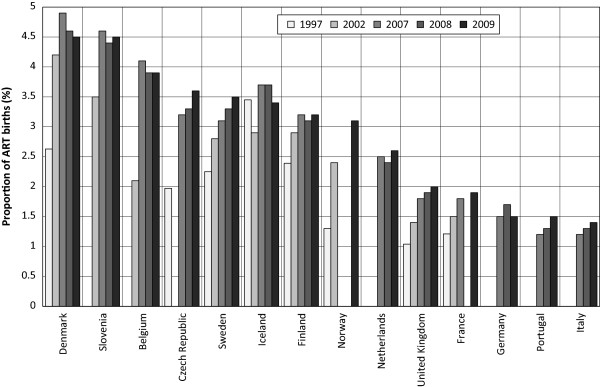
**Trends in percentage of ART births between 1997 and 2009 in selected European countries.** Data sources: ESHRE, The Czech National ART Register*.*

Figure 1 presents ART use in countries which provided complete data at least during three years within the period under observation. Between 1997 and 2007, all selected countries except Iceland, experienced a continuous increase in the percentage of live births following the use of ART. However, due to an unequal rate of increase in ART use across countries, cross-country variation deepened. The highest increase in percentage of live births following the use of ART was found in Denmark, Belgium, Slovenia, and the Czech Republic. The highest proportion of children born following ART was reached in Denmark (close to 5%) in 2007. The year 2007 can be taken as a turning point particularly for countries with a proportion exceeding 3.5%: up until 2009, all of the latter recorded a decrease or stabilization at around the level of 4.5%, suggesting that a possible threshold was reached. An uninterrupted increase in the proportion of children born following ART was only found in a few countries with a lower percentage such as Italy (1.4% in 2009), France (1.9% in 2009) and the United Kingdom (2% in 2009) as well as Sweden (3.5% in 2009) and Norway (3.1% in 2009). Recently the percentage of children born following ART exceeded 3% in Northern European countries, Belgium, the Czech Republic and Slovenia.

### Relationship between ART use and the fertility rate

**Figure 2:**
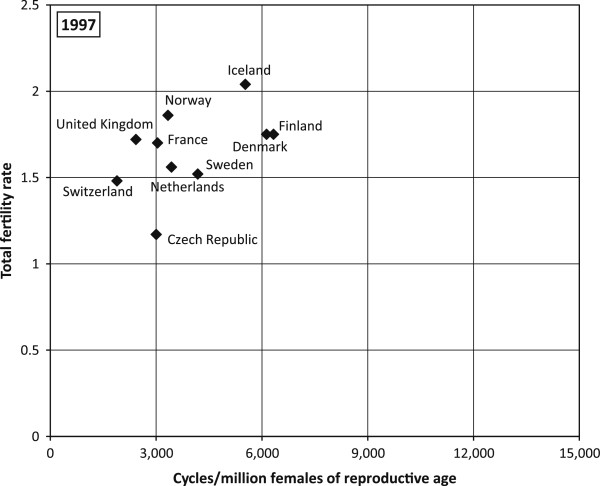
**European countries by TFR and ART cycles per million women aged 15–49, 1997.** Data sources: ESHRE, Eurostat*.*

**Figure 3:**
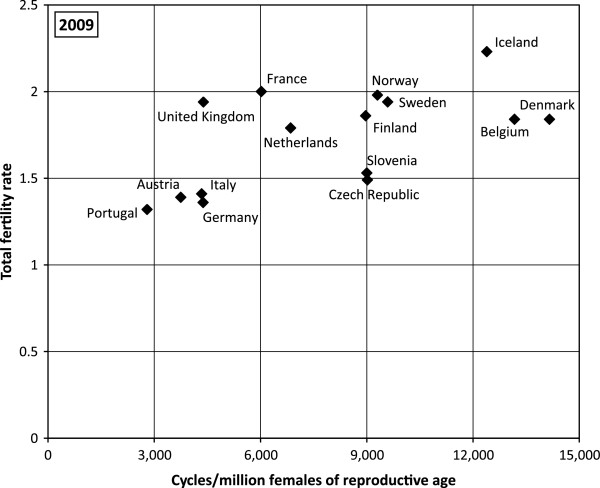
**European countries by TFR and ART cycles per million women aged 15–45, 2009.** Data sources: ESHRE, Eurostat*.*

In 1997, countries with a higher TFR reported a higher number of treatment cycles per 1 million women of reproductive age (see Figure 2). By 2009, the number of treatment cycles had doubled specifically in countries that registered TFR close to 2 children per woman (Figure 3). Furthermore a substantial increase in ART cycles was also relevant for countries with a low TFR, i.e. the Czech Republic and Slovenia. In contrast to 1997, in 2009 the Czech Republic and Slovenia reported a higher number of treatment cycles per 1million women aged 15–45 than France and the Netherlands. Consequently, both the Czech Republic and Slovenia experienced a significant increase in TFR between 1997 and 2009 and the plot did not vary significantly until 2009 as most countries with a higher TFR registered a higher number of treatment cycles per 1million women of reproductive age as well.

### Trends in age structure of women treated with ART

The countries under study significantly differ according to the age structure of women who requested IVF/ICSI (Table 1). Variations even increased between 1997 and 2009 as the proportion of women aged 40 and over ranged between 9% in the Czech Republic and 15% in Switzerland in 1997, while in 2009 it was between 7% in the Czech Republic and 28% in Italy. Interestingly, a rather significant proportion of women who requested IVF was constituted by younger women, younger than 35. In the Czech Republic, women younger than 35 represented almost 70% of the total number who had requested ART. Moreover, in the Czech Republic there was almost no change in the age structure of women who had requested IVF/ICSI between 1997 and 2007. On the contrary, most other countries in Table 1, i.e. Italy, Switzerland, Spain, Sweden, Germany, Portugal, and Hungary, did register quite a significant increase in the share of women aged 35 and over. Recently the highest share of women aged 35 and over was found in Ireland and Italy (almost 70%). In countries with the highest proportion of ART births, i.e. Denmark or Slovenia, women aged 35 and over accounted for less than 50% of the total number who had requested ART.

### Relationship between the use of ART and birth timing

**Figure 4:**
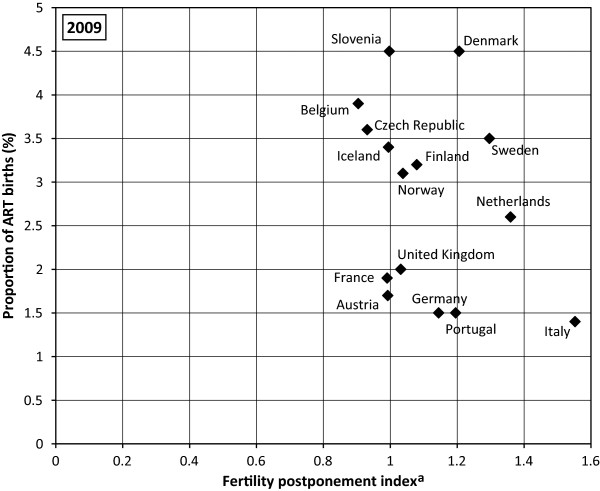
**European countries by percentage of ART births and fertility postponement index, 2009. **^a^ Lesthaeghe & Niedert´s fertility postponement index (FPI) is the ratio of the sum of ASFR above age 29 to the sum of these rates between ages 20 and 29. Data sources: ESHRE, Eurostat, The Czech National ART Register*.*

**Figure 5:**
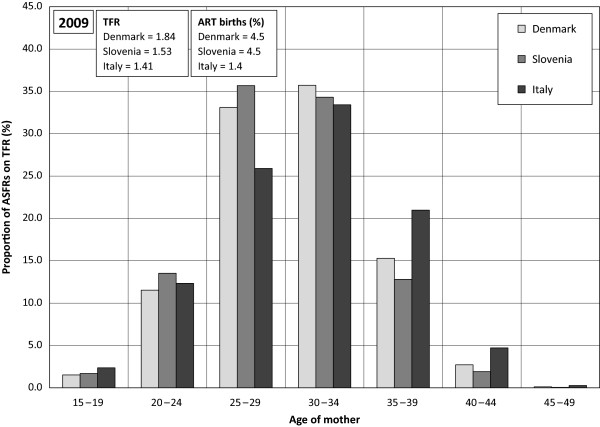
**Proportion of ASFRs on TFR in%, Denmark, Slovenia, Italy, 2009.** Data source: Eurostat*.*

**Figure 6:**
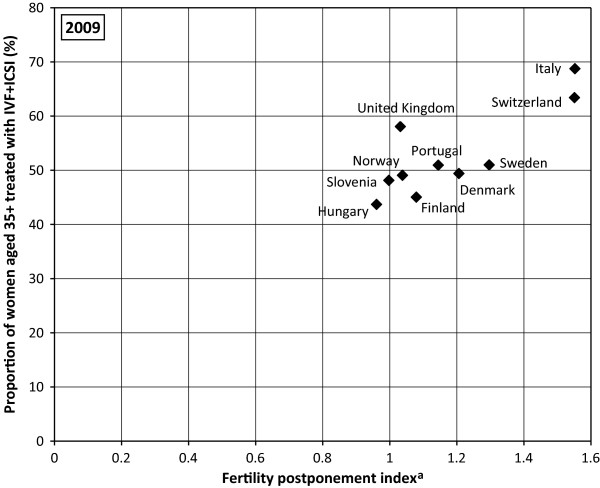
**European countries by percentage of women 35+ treated with IVF/ICSI and fertility postponement index, 2009. **^a^ Lesthaeghe & Niedert´s fertility postponement index *(FPI) is the ratio of the sum of ASFR above age 29 to the sum of these rates between ages 20 and 29. Data sources: ESHRE, Eurostat.*

No direct link between the percentage of children born following ART and the postponement rate in countries under study was found (Figure 4). Actually the highest percentage of children born following ART was found in countries with a less advanced postponement rate, i.e. with FPI between 1 and 1.2 (Slovenia and Denmark). Furthermore, the highest postponement rate registered in Italy has not been reflected in a high percentage of children born following ART but the reverse is true. Figure 5 gives a more detailed picture regarding fertility age schedules in these three countries. A high percentage of children born following ART and a high TFR in Denmark are connected with fertility schedules concentrated on women aged between 25 and 34, i.e. with a “broad peak” fertility pattern. Both Denmark and Slovenia have experienced a sharp rise of fertility in early 20s and late 20s as well as a sharp decrease in fertility from 34 onward. On the contrary, a low proportion of ART births and a low TFR are connected to a “delayed” fertility pattern. Accordingly, an effective ART use can be expected in countries with “broad peak” fertility schedules rather than in countries with a “delayed” fertility schedule. The increasing demand for ART is significant among women who delay childbearing and are by definition older. The positive relationship existing – scale between the postponement rate and the percentage of women aged 35 and older treated with IVF/ICSI, was definitely established in countries under study (Figure 6).

## Discussion

We found out that though ART use is widespread in countries under study, large-scale differences in its use still prevail. The sharp increase in the number of treatment cycles, particularly between 1997 and 2007, reflected an increasing demand for ART. In most countries the increase in the proportion of ART births was in relation to the increase in TFR [32]. Accordingly, we suppose that recent fertility recuperation trends led to the rising need of ART treatment. Nevertheless, countries significantly differed as to the rate of increase within the proportion of ART births.

The important determinant of ART use is state health insurance policy [11]. Although the impact of reimbursement on ART use was not the primary aim of our study, differences in the range of public insurance coverage among countries under study, have to be taken into account. Denmark and Belgium seem to be leading as to health insurance reimbursement patterns: ART in Denmark is provided free of charge to women below the age of 40, up to three cycles and is easily accessible at public clinics [33]. In Belgium even up to six ART cycles are reimbursed to women under 43 [34]. Thus both countries registered a substantial increase in the proportion of ART births by 2007. On the contrary, Germany has denoted the negative impact of a more restrictive reimbursement policy introduced in 2004 [35]: co-payments of ART treatment were raised for childless couples and the number of subsidized treatments limited to three. As a result, Germany has remained among countries with a low ART use. ART use demographic potential seems to become increasingly dependent on state supportive policies of ART Thus at the level of ART affordability to the public. This has become particularly evident since 2008 when due to the economic recession, massive cuts in public spending reduced state family-related expenditures and consequently also fertility decision making [36]. A discontinuation of the increase in the proportion of ART births was registered particularly in countries where the previous TFR upturn had been replaced by its stagnation or decline, i.e. in Denmark, Slovenia, Belgium, Finland and Germany. While in Denmark or Belgium a threshold in the use of ART was probably reached prior to 2008, other countries such as Germany had not yet fully utilized ART potential and a stimulation of the increase in ART use would need additional investments.

Our results indicate that the growing utilization of ART has become relevant when assessing recent fertility trends. Some recent studies documented that the potential contribution of ART to rising fertility rates was not negligible [37-40]. Despite the limits in the increase of ART use, its impact on the fertility level could be significant particularly in countries with TFR below 1.5 [41] although most studies overestimated the effectiveness of ART or neglected biological and behavioural factors when assessing its true effect [10], It is suggested that ART support become an integral part of national strategies addressing demographic and reproductive challenges [42]. However in reality, it is clearly exceptional since only Denmark’s reimbursement scheme has been influenced by demographic concerns so far [43]. An explicit ART policy based on goals different from demographic ones may be more acceptable and more effective. Implicitly, an enhancement of fertility could be expected. Indeed, taking Belgium as an example, a well-founded strategy meant to improve access to ART treatment based on the aim of supporting the birth of a healthy singleton child, could have a demographic impact.

A considerable change in birth timing is the key demographic aspect related to ART use which then, may go against the endeavour to enhance state support to ART. It was argued that ART increased availability might create a false perception among the public that childbearing could be postponed until late reproductive age groups - ART making pregnancy possible for almost any prospective mother [40]. Our results do suggest that the increasing prevalence of women aged 35 and over among all women who asked for IVF/ICSI, has been undoubtedly part of the effect of childbearing postponement. However, the age structure of women treated with IVF/ISCI is younger in Denmark than in the United Kingdom although ART is more subsidized in Denmark. Instead, ART better availability might encourage couples to seek help sooner rather than later.

Nevertheless, besides ART reimbursement policy additional factors have to be taken into account when explaining differences in age structures of women treated with IVF/ICSI. Firstly, guidelines related to “the waiting period” before applying for IFV when attempts to conceive naturally failed, may vary from one year in the Czech Republic [44] to three years in the Netherlands [45]. Secondly, the number of cycles and the female age limit for reimbursement can play a role within the well-timed decision making process. Probably, the low number of reimbursed cycles coupled with the female low age limit as it is in the Czech Republic - up to three cycles until the age of 39 [44] - would stimulate women to ask for an ART treatment earlier in their lifetime so as to have a better chance at succeeding, compared to Belgium where women under 43 are entitled up to six reimbursed cycles [43]. Accordingly the Czech Republic has maintained the youngest female age structure treated with IVF/ICSI despite the trend towards postponement.

While increased ART use is considered to be the result of childbearing postponement, we found out that it need not contribute to delayed childbearing, i.e. to the increased mean age of mothers at childbirth. Moreover, trends towards a rectangularization of fertility, i.e. reduction of variability of mothers’ age at first birth and concentration of most births around mothers’ higher age, have not been discerned [46]. Our results indicated that the demographic relevancy of ART use is more common in a population with a less advanced postponement rate. It supports previous findings about the importance of ART contribution to fertility rates of women currently in the middle of their reproductive span [40]. Although the increase in the proportion of women aged 35 and over among those who requested ART was established, the relevant higher fertility rates of women aged 35 and over were not apparent. As ART success rate dramatically decreases with women aged 35 and over, an increasing ART use does not significantly contribute to the increase in female age at childbirth. A more intense childbearing postponement could probably develop if ART success rates significantly improve among late childbearing age groups. Currently, increased ART use is expected to have a demographic impact if women take advantage of it earlier rather than later in life. Moreover, women who may have been able to conceive with a less aggressive therapy, may request ART earlier in states with a comprehensive insurance policy [47].

Although we want to contribute to a better understanding of demographic relevancy of increased ART use, we are definitely aware of the limits of our findings as we focus exclusively on ART. A fertility increase may be the result of diverse types of infertility treatments. In contrast to our results, the implementation of insurance coverage of infertility treatment including IVF in the US, increased first birth rates for women 35 and older [48]. However, findings are not fully comparable particularly due to the use of different data. While we concentrated on studying the relationship between ART use and fertility trends from a cross-country perspective, in the US the effects of insurance mandates on fertility were analysed for the period 1981–1999 using data on births. Moreover, women who are older and highly educated are more likely to be affected by mandates due to their higher probability of having private health insurance [48].

## Conclusions

Findings for some European countries suggest that increased ART use may have a demographic relevancy when women take advantage of it earlier rather than later in life. ART widespread use may become one of the factors keeping the fertility rate stable in the future regardless of negative trends. Accordingly, it is suggested that the use of ART at a younger age should be supported so as to increase the chance of women to achieve their reproductive goals while reducing the risk of age-related infertility and failed ART. An ART reimbursement policy could be part of a reproductive health policy promoting early childbearing: it could keep women from delaying having children and increase the chance of diagnosing potential reproductive health problems requiring a timely ART application.

## Abbreviations

ART: Assisted reproduction technology; TFR: Total fertility rate; ASFR: Age specific fertility rate; FPI: Fertility postponement index; EU: European Union; EIM: European IVF-monitoring; ESHRE: European Society of Human Reproduction and Embryology; IVF: In Vitro fertilization; ICSI: Intracytoplasmic sperm injection; FER: Frozen embryo replacement; ED: Oocyte donation; IVM: In Vitro maturation; PGD: Preimplantation genetic diagnosis; FOR: Frozen oocyte replacement.

## Competing interests

The authors declare that they have no competing interests.

## Authors’ contributions

JK conceptualized the study, wrote the main body of the paper, contributed to the research, data analysis and editing. BB was involved in the research, methodological design, data analysis and editing of the paper. TK contributed to the research and data analysis. All authors have read and approved the final version of the paper.

## References

[B1] Kohler HP Billari FC Ortega JA The emergence of lowest-low fertility in Europe during the 1990s Popul Dev Rev 2002 28 641 681 10.1111/j.1728-4457.2002.00641.x

[B2] Frejka T Sobotka T Fertility in Europe: diverse, delayed and below replacement Demographic Res 2008 19 15 45

[B3] Goldstein JR Sobotka T Jasilioniene A The end of lower-low fertility? Popul Dev Rev 2009 35 663 700 10.1111/j.1728-4457.2009.00304.x

[B4] Beets G A contemporary issue in demography: The rising age at first birth, pros and cons AUC Geographica 2011 46 5 14

[B5] VID Vienna Institute of Demography, Austrian Academy of Sciences, and International Institute for Applied Systems Analysis (IIASA) European Demographic Data Sheet 2012 2012 Available from http://www.oeaw.ac.at/vid/datasheet/index.html

[B6] Hughes EG Giacomini M Funding in vitro fertilization treatment for persistent subfertility: the pain and the politics Fertil Steril 2001 76 431 442 10.1016/S0015-0282(01)01928-8 11532460

[B7] Broekmans FJ Knauff EAH te Velde ER Macklon NS Fauser BC Female reproductive ageing: current knowledge and future trends Trends in Endocrinol Metabol 2008 18 58 65 10.1016/j.tem.2007.01.004 17275321

[B8] Leridon H Can assisted reproduction technology compensate for the natural decline in fertility with age? A model assessment Hum Rep 2004 19 1549 1554 10.1093/humrep/deh304 15205397

[B9] Sobotka T Zeman K Lesthaeghe R Frejka T Neels K Postponement and Recuperation in Cohort Fertility: Austria, Germany and Switzerland in a European context Comp Popul Stud 2011 36 417 452

[B10] ESHRE Capri Workshop Group Europe the continent with the lowest fertility Hum Reprod Update 2010 16 590 602 20603286 10.1093/humupd/dmq023

[B11] Connolly MP Hoorens S Chambers GM on behalf of the ESHRE Reproduction and Society Task Force The costs and consequences of assisted reproductive technology: an economic perspective Hum Reprod Update 2010 16 603 613 10.1093/humupd/dmq013 20530804

[B12] Ferraretti AP Goossens V Kupka M Bhattacharya S de Mouzon J Castilla JA Erb K Korsak V Andersen AN Assisted reproductive technology in Europe, 2009: results generated from European registers by ESHRE Hum Rep 2013 28 2318 2331 10.1093/humrep/det278 23842560

[B13] Chambers GM Sullivan EA Ishihara O Chapman MG Adamson GD The economic impact of assisted reproductive technology: a review of selected developed countries Fertil Steril 2009 91 2281 2294 10.1016/j.fertnstert.2009.04.029 19481642

[B14] Jones HW Allen BD Strategies for designing an efficient insurance fertility benefit: a 21^st^ century approach Fertil Steril 2009 91 2295 2297 10.1016/j.fertnstert.2008.03.006 18440518

[B15] Schmidt L Andersen AN What is a baby-friendly policy? Is everything there? Pharmaceuticals Policy Law 2007 9 69 76

[B16] ESHRE Capri Workshop Group Social determinants of human reproduction Hum Rep 2001 16 1518 1526 10.1093/humrep/16.7.1518 11425841

[B17] Dawson AA Diedrich K Felberbaum RE Why do couples refuse or discontinue ART? Arch Gynecol Obstet 2005 273 3 11 10.1007/s00404-005-0010-5 16080011

[B18] Leridon H Slama R The impact of a decline in fecundity and of pregnancy postponement on final number of children and demand for ART Hum Rep 2008 23 1312 1319 10.1093/humrep/den106 18387960

[B19] Mills M Rindfuss RR McDonald P te Velde E on behalf of the ESHRE Reproduction and Society Task Force Why do people postpone parenthood? Reasons and social policy incentives Hum Reprod Update 2011 17 848 860 10.1093/humupd/dmr026 21652599 PMC3529638

[B20] Te Velde E Habbema D Leridon H Eijkemans M The effect of postponement of first motherhood on permanent involuntary childlessness and total fertility rate in six European countries since the 1970s Hum Rep 2012 27 1179 1183 10.1093/humrep/der455 22258662

[B21] Schmidt L Sobotka T Bentzen JG Andersen AN on behalf of the ESHRE Reproduction and Society Task Force Demographic and medical consequences of the postponement of parenthood Hum Reprod Update 2012 18 29 43 10.1093/humupd/dmr040 21989171

[B22] Nygren KG Andersen AN Assisted reproductive technology in Europe, 1997. Results generated from European registers by ESHRE Hum Rep 2001 16 384 391 10.1093/humrep/16.2.384 11157839

[B23] Andersen AN Gianaroli L Felberbaum R de Mouzon J Nygren KG Assisted reproductive technology in Europe, 2002. Results generated from European registers by ESHRE Hum Rep 2006 21 1680 1697 10.1093/humrep/del075 16585126

[B24] De Mouzon J Goossens V Bhattacharya S Castilla JA Ferraretti AP Korsak V Kupka M Nygren KG Andersen AN Assisted reproductive technology in Europe, 2006: results generated from European registers by ESHRE Hum Rep 2010 25 1851 1862 10.1093/humrep/deq124 20570973

[B25] De Mouzon J Goossens V Bhattacharya S Castilla JA Ferraretti AP Korsak V Kupka M Nygren KG Andersen AN Assisted reproductive technology in Europe, 2007: results generated from European registers by ESHRE Hum Rep 2011 26 954 966 10.1093/humrep/des023 PMC3303494 22343707

[B26] Ferraretti AP Goossens V de Mouzon J Bhattacharya S Castilla JA Korsak V Kupka M Nygren KG Andersen AN Assisted reproductive technology in Europe, 2008: results generated from European registers by ESHRE Hum Rep 2012 27 2571 2584 10.1093/humrep/des255 22786779

[B27] Zegers-Hochschild F Adamson GD de Mouzon J Ishihara O Mansour R Nygren K Sullivan E Vanderpoel S on behalf of ICMART and WHO The International Committee for Monitoring Assisted Reproductive Technology (ICMART) and the World Health Organization (WHO) Revised Glossary on ART Terminology Hum Rep 2009 24 2683 2687 10.1093/humrep/dep343 19801627

[B28] Eurostat Populations and Social Conditions 2013 Available from http://epp.eurostat.ec.europa.eu

[B29] Nations World Population Prospects: The 2010 Revision 2011 New York: United Nations, Population Division

[B30] Lesthaeghe RJ Neidert L The Second Demographic Transition in the United States: Exception or Textbook Example? Popul Dev Rev 2006 32 1 31

[B31] Lesthaeghe RJ Lopez-Gay A Spatial continuities and discontinuities in two successive demographic transitions: Spain and Belgium, 1880–2010 Demographic Res 2013 28 77 136

[B32] Luci A Thévenon O Does economic development explain the fertility rebound in OECD countries? Popul Societies 2010 481 1 4

[B33] Andersen AN Erb K Register data on ART in Europe including a detailed description of ART in Denmark Intern J of Andrology 2006 29 12 16 10.1111/j.1365-2605.2005.00577.x 16466519

[B34] Ombelet W Access to assisted reproduction services and infertility treatment in Belgium in the context of the European countries Pharmaceuticals Policy Law 2007 9 189 202

[B35] Ochel W Osterkamp R Fertility policy in Germany Pharmaceuticals Policy Law 2007 9 211 219

[B36] Sobotka T Skirbekk V Philipov D Economic recession and fertility in the developed world Popul Dev Rev 2011 37 267 306 10.1111/j.1728-4457.2011.00411.x 22066128

[B37] Sunde A Europe´s declining population and the contribution of ART Pharmaceuticals Policy Law 2007 9 79 90

[B38] Grant JC Hoorens S Gallo F Cave JAK Should ART be part of a population mix? A preliminary assessment of the demographic and economic impact of assisted reproductive technologies 2006 The RAND Corporation Available from http://www.rand.org/content/dam/rand/pubs/documented_briefings/2006/RAND_DB507.pdf 10.1093/humrep/dem181 17586831

[B39] Hoorens S Gallo F Cave JAK Grant JC Can assisted reproductive technologies help to offset population ageing? An assessment of the demographic and economic impact of ART in Denmark and UK Hum Rep 2007 22 2471 2475 10.1093/humrep/dem181 17586831

[B40] Sobotka T Hansen MA Jensen TK Pedersen AT Lutz W Skakkebaek NE The contribution of ART to completed fertility: an analysis of Danish data Popul Dev Rev 2008 34 79 101 10.1111/j.1728-4457.2008.00206.x

[B41] Kocourkova J Fait T Can increased use of ART retrieve the Czech Republic from the low fertility trap? Neuroendocrinol Lett 2009 30 111 118 20038934

[B42] Ziebe S Devroey P on behalf of the State of the ART 2007 Workshop Group Assisted reproductive technologies are an integrated part of national strategies addressing demographic and reproductive challenges Hum Reprod Update 2008 14 583 592 10.1093/humupd/dmn038 18786951

[B43] ESHRE Comparative Analysis of Medically Assisted Reproduction in the EU:Regulation and Technologies 2008 SANCO/2008/C6/051

[B44] Kocourkova J Burcin B Demografická specifika asistované reprodukce v České republice v evropském kontextu Demografie 2012 54 250 263

[B45] Habbema JDF Eijkemans MJC Nargund G Beets G Leridon H te Velde ER The effect of in vitro fertilization on birth rates in western countries Hum Rep 2009 24 1414 1419 10.1093/humrep/dep004 19233869

[B46] Billari FC Kohler HP Andersson G Lundström H Approaching the limit: long-term trends in late and very late fertility Popul Dev Rev 2007 33 149 170 10.1111/j.1728-4457.2007.00162.x

[B47] Henne MB Bundorf MK Insurance mandates and trends in fertility treatments Fertil Steril 2008 89 66 73 10.1016/j.fertnstert.2007.01.167 17482603

[B48] Schmidt L Effects of infertility insurance mandates on fertility J Health Econ 2007 26 431 446 10.1016/j.jhealeco.2006.10.012 17129624 PMC2096618

